# Complete Molecular Response in Chronic Myeloid Leukemia After Six Months of Imatinib: A Single Center Experience

**DOI:** 10.7759/cureus.7826

**Published:** 2020-04-25

**Authors:** Umera Saleem, Talha Hafeez, Syed Ali Raza, Muhammad Tahir, Fatima Khalid, Muhammad Khurrum Islam

**Affiliations:** 1 Pathology, Nishtar Medical University & Hospital, Multan, PAK; 2 Oncology, Jinnah Hospital, Lahore, PAK

**Keywords:** chronic myeloid leukemia, imatinib

## Abstract

Introduction: The hallmark of chronic myeloid leukemia (CML) is the development of the fusion gene, BCR-ABL which has unopposed tyrosine kinase activity. The first tyrosine kinase inhibitor (TKI) imatinib is claimed to have superior efficacy and side effect profile as compared to traditional treatment options. This study was conducted to see our patients’ molecular response to imatinib treatment. The objective of this study was to determine the frequency of complete molecular response in patients after six months of imatinib therapy.

Methods: A descriptive case series was designed and conducted in Oncology department, Jinnah hospital Lahore (May-November 2016). Newly diagnosed patients of CML aged between 20 and 65 years were enrolled. They were prescribed 400 mg imatinib daily and complete molecular response was assessed after six months of treatment.

Results: Mean age was 39.76 ± 9.072 years. Some 66 of them were males while 69 were females. Some 40 patients (29.6%) were found to be in complete molecular response after six months of imatinib therapy.

Conclusion: Imatinib at a dose of 400 mg/day is optimal as the primary therapy for CML.

## Introduction

Chronic myeloid leukemia (CML) is a myeloproliferative neoplasm in which translocation between chromosome 9 and 22 leads to the development of a hybrid chromosome 22 called as Philadelphia chromosome. The underlying molecular defect is a fusion gene called BCR-ABL which encodes the oncoprotein BCR-ABL1 (also referred to as p210, p190, p230) with a constitutive active tyrosine kinase activity [1-2].

The annual incidence rate of CML is 0.7-1.0 per 100,000 people [3]. The natural course of the disease is triphasic: a chronic phase (CP), an accelerated phase (AP), and a blast crisis (BC). The majority of patients are diagnosed in the CP [4].

 Tyrosine kinase inhibitor (TKI) therapy has promising response rates in CML [5]. Imatinib mesylate was the prototype drug approved by FDA in 2001. This has revolutionized the treatment of CML from control towards cure [6]. This study was conducted to document response rates in our patients to standard dose of imatinib.

## Materials and methods

A descriptive case series was conducted in the Oncology department, Jinnah Hospital, Lahore from 24th May 2016 to 23rd November, 2016. A sample size of 135 cases was calculated with 95% confidence level, 8% margin of error, and considering expected frequency of complete molecular response as 34%. Patients of both genders having age range between 20 and 65 years of CML were selected as the study population. Patients with prior treatment for CML or those having serological evidence of infection by human immunodeficiency virus were excluded.

Approval of hospital ethical review committee was taken and anonymity of data was maintained. After taking written informed consent, diagnostic and baseline tests were performed at presentation. All patients were prescribed imatinib at a dose of 400 mg daily for six months. Molecular response was assessed after six months of treatment and monitored by FISH analysis on peripheral blood sample.

The data were analyzed using SPSS version 20. Mean and standard deviation were calculated for quantitative variables such as age. Qualitative variables such as gender and complete molecular response were expressed as frequencies. Effect modifiers such as age and gender were controlled by stratification. Poststratification chi-square test was applied and p value ≤ 0.05 was taken as significant.

## Results

A total of 135 cases were recruited in the study. Mean age of the patients was 39.76 ± 9.0 years with an age range between 24 and 65 years. Only 7.4% were younger (<35 years). Female gender constituted 51.1% of total patients.

Splenomegaly was seen in 86% and Philadelphia positivity in 98.9%. Characteristics of study population are shown in Table 1.

Sokal score of our patients was as follows: 6%, 30%, and 64% in low risk (LR), intermediate risk (IR), and high risk (HR) category respectively.

**Table 1:** Characteristics of study population.

Characteristics	Baseline	Post six months	p value
Hb	11.8 ± 3.5	10.5 ± 1.8	0.0001
TLC	30.73 ± 5.38	21.32 ± 5.91	0.0001
Platelet count	405.5 ± 280	316.0 ± 155.5	0.0001
Basophils	19.2 ± 4.39	19.2 ± 5.55	0.0001
Blasts	10.41 ± 1.68	6.04 ± 3.94	0.0001
Philadelphia chromosome positivity	98.9 ± 20.68	23.37 ± 19.5	0.0001

Some 40 patients (30%) fulfilled the criteria of complete molecular response after six months of imatinib therapy. Among age groups, older patients and female gender achieved complete molecular response than young patients and male gender (Table 2).

**Table 2:** Distribution of complete molecular response according to age and gender.

Complete molecular response	Yes	No	p value
	Number (percentage)	Number (percentage)	
Overall			
	40 (30)	95 (70)	0.0321
According to age groups			
<35 years	04 (40)	06 (60)	0.0452
35-50 years	19 (33.3)	38 (66.7)	
>50 years	17 (66.7)	51 (75)	
According to gender			
Male	14 (21.2)	52 (78.78)	0.024
Female	20 (29)	49 (71)	

## Discussion

Chronic myeloid leukemia is a clonal disorder which leads to uncontrolled proliferation of myeloid cells. The disease generally has an indolent course and remains stable for years. In few patients, however, it can transform to an AP or a blast phase with a poor outcome.

The disease can manifest at any age but generally is believed to be a disease after fifth decade of life. Mean age of our patients was 39.76 years. This is similar to other studies conducted in Southeast Asia but lower than compared to Western countries such as Sweden where mean age of presentation is reported to be 60 years [7-9]. The difference could be attributed to demographic characteristics of developing countries or genetic factors.

Female gender was more common than males in our study (51.1%). This was unusual as CML is reported to be more common in males [10].

None of the patients was asymptomatic and moderate to massive splenomegaly was seen in 86% of our patients. Our findings are comparable to a Nigerian study where 91.2% patients had splenomegaly [11].

Philadelphia chromosome is a constant feature of CML seen in more than 90% of patients [4]. It was positive in 98.9% of our patients at diagnosis with BCR-ABL fusion gene in 100% patients.

Out of 135 patients, 6% of our patients were in LR category according to Sokal score, 30% in IR category, and 64% in HR group. These are comparable to a Pakistani study by Usman et al. which quotes 3.7%, 27.4%, and 67.7% respectively [12]. However the pattern reported by a study in Nigeria is different compared to the present study results (LR-40.3%, IR-33.6%, and HR-26.1%) [13].

Complete molecular response was seen in 30% of our patients after six months of 400 mg imatinib therapy (p value 0.0321). Our results are in concordance to a randomized CELSG phase III CML 11 “ISTAHIT” study where 34% patients achieved complete molecular response [14]. Another phase III randomized, open-label study of daily imatinib 400 mg versus 800 mg in CML found that the 20% patients taking standard dose of imatinib had complete molecular response and that safety and efficacy of both doses were equal [15]. While in ELN study, the response on standard dose was 32% [16].

The response to imatinib in >50-65 years age group was 66.7%. This is comparable to a Swedish study where it was 79% [17].

More females (29%) responded to imatinib than males (20%). This difference was statistically significant. Our results are similar to those of Lin et al. [18].

## Conclusions

Imatinib induces complete molecular response in CML especially in older patients and females after six months of therapy. Therefore, imatinib at a dose of 400 mg/day should be considered as optimal primary treatment for CML.
